# Indications, Management, and Short- and Medium-Term Outcomes of Patients with Chronic Coronary Occlusion Treated with Percutaneous Revascularization—A Single-Center Study

**DOI:** 10.3390/jcdd12020075

**Published:** 2025-02-16

**Authors:** Lucia Barbieri, Gabriele Tumminello, Lorenzo Mafrici, Guido Pasero, Luca Mircoli, Federico Colombo, Cecilia Gobbi, Alessandra S. Rizzuto, Stefano Carugo

**Affiliations:** 1 Department of Cardio-Thoracic-Vascular Diseases, Foundation IRCCS Ca’ Granda Ospedale Maggiore Policlinico, 20122 Milan, Italy; tumminellogabriele@gmail.com (G.T.); luca.mircoli@policlinico.mi.it (L.M.); federico.colombo@policlinico.mi.it (F.C.); cecilia.gobbi@policlinico.mi.it (C.G.); stefano.carugo@unimi.it (S.C.); 2 Department of Clinical Sciences and Community Health, University of Milan, 20122 Milan, Italy; loremafrici31@gmail.com (L.M.); guido.pasero@gmail.com (G.P.); alessandra.rizzuto@unimi.it (A.S.R.)

**Keywords:** chronic total occlusion, coronary intervention, patient outcome

## Abstract

The diagnosis of chronic total occlusion (CTO), characterized by the complete obstruction of a coronary artery for at least three months, remains challenging and can be entirely asymptomatic. Since the indications for performing a recanalization procedure for CTO do not originate from randomized controlled trials, this study aimed to assess the indications, management, and procedural outcomes of patients undergoing percutaneous revascularization (PCI) for a CTO, ensuring that the population was as uniform as possible regarding technologies and methodological approaches. Forty-one consecutive patients who underwent PCI for CTO recanalization were enrolled from January 2021 to 2024. Additional outcomes included mortality, major adverse cardiovascular events, and the presence of residual cardiac symptoms, with a median follow-up of 449 days and an interquartile range of 230–643 days. Our real-life study confirmed that PCI for CTO has a high success rate and a low incidence of major complications.

## 1. Introduction

Chronic total occlusions (CTOs) represent a challenging subset of coronary artery disease (CAD), defined by the complete obstruction of a coronary artery for at least three months. The exact epidemiology in the general population is unknown, as diagnosis requires angiographic investigation, and CTOs can present completely asymptomatically. The prevalence ranges from 10% in non-culprit vessels in patients undergoing primary PCI for acute ST-elevation myocardial infarction (STEMI) to 54% in patients who have undergone coronary artery bypass graft surgery [1]. In CTOs, the varying composition of vascular obstruction is related both to the mechanism of occlusion formation and to the duration of the occlusion itself [2]. The presence of calcifications typically ranges from small and partial calcifications in short-duration CTOs to significant areas of calcification, commonly found in long-duration CTOs, the extent of which can significantly influence procedural success. In CTOs, there is also an extensive process of neoangiogenesis, with the formation of neovessels that traverse the plaque. These vessels typically reach the size of large capillaries (100–500 μm) and can be categorized into two types: intraluminal microchannels and ipsilateral bridging collaterals. The former may be traversable by a guidewire, potentially playing an active role in revascularization strategies [3]. Conversely, collateral vessels are defined as vascular connections that link parallel arteries without the interference of a capillary bed, allowing blood flow in the presence of coronary obstruction. They may originate from the same occluded vessel, termed homocoronary collaterals, or from the contralateral vessel, termed heterocoronary collaterals. Identifying the presence of collateral vessels is very useful during CTO PCI, as it allows the visualization of the coronary segment distal to the occlusion [4]. The likelihood of the successful revascularization of a CTO primarily depends on anatomical factors and the intrinsic characteristics of the lesion. Based on these characteristics, various scores have been developed to quantify the probability of successful CTO recanalization [5]. The J-CTO score is the most commonly used: by considering five different variables (cap shape, calcifications, curvature (bending) > 45°, occlusion length ≥20 mm, and whether there has been a previous failed attempt at recanalization), it calculates a score (from 0 to 3) that determines the anticipated level of difficulty of the procedure [6]. CTO revascularization is highly technically complex and is therefore performed in a staged manner following adequate pre-procedural planning. CTO-PCI techniques are divided into antegrade and retrograde approaches, with several different techniques developed depending on local expertise, patient selection, and material availability. The anterograde approach is the primary strategy in approximately 70% of cases due to its lower risk of periprocedural complications; specifically, anterograde wire escalation (AWE) is the most frequently used technique [7]. The indications for performing a recanalization procedure for CTO do not originate from randomized controlled trials but predominantly from observational studies and are therefore related to individual centers or working groups that have applied different criteria. In 2018, the European Society of Cardiology (ESC) [8] addressed this issue, stating that PCI for a CTO should be considered in patients with angina refractory to medical therapy or in the presence of a large ischemic area documented in the territory supplied by the occluded vessel (Class IIa, Level of Evidence B), similarly to conventional PCI in patients with chronic coronary syndromes. An analysis of the literature [9] has shown a reduction in mortality in patients successfully recanalized compared to those in whom the procedure was unsuccessful. Conversely, randomized studies comparing CTO-PCI and medical therapy have not demonstrated a reduction in mortality with the invasive strategy [10,11,12,13,14]. Additionally, it should be noted that CTO-PCI procedures are often complex and not free from complications, even in the hands of highly experienced operators. Based on these premises, the aim of this study was to evaluate the indications, management, and procedural outcomes of patients treated with the percutaneous revascularization of a CTO. An additional objective was to assess patient outcomes at follow-up regarding mortality, major adverse cardiovascular events (MACEs), and the presence of residual cardiac symptoms.

## 2. Materials and Methods

We conducted a retrospective analysis of patients who underwent percutaneous revascularization for the recanalization of a CTO from January 2021 to January 2024 at the Hemodynamics Laboratory of the Fondazione IRCCS Ca’ Granda Ospedale Maggiore Policlinico in Milan. Data were collected in a dedicated password-protected database, and all patients signed an informed consent form regarding the treatment of personal data. Regarding the clinical characteristics of the population, hypertension was defined as a systolic blood pressure > 140 mmHg and/or a diastolic blood pressure ≥ 90 mmHg or the presence of antihypertensive therapy at the time of admission. Diabetes mellitus was defined as a fasting blood glucose > 126 mg/dL, a random blood glucose > 200 mg with associated symptoms, a glycosylated hemoglobin > 6.5%, or the presence of specific therapy upon admission. The presence of angina was defined as chest pain on exertion of likely cardiac origin or the presence of equivalent angina symptoms (dyspnea, reduced functional capacity). Instrumental ischemia was assessed through the execution of cardiovascular stress tests or stress imaging studies. The presence of viability in the territory supplied by the target vessel was evaluated using cardiac magnetic resonance or a pharmacological stress echocardiogram with a low dose of dobutamine. All patients were treated according to current guidelines [8] regarding procedural management and inpatient care. Follow-up was performed by telephone if the last available reassessment was > 6 months prior. Death, readmission for cardiovascular issues, the revascularization of the target vessel, stroke, acute myocardial infarction, and the persistence of residual angina symptoms were recorded as adverse events during follow-up. The presence of MACEs at follow-up was defined as the occurrence of at least one of the following: readmission for cardiovascular issues, the revascularization of the target vessel, stroke, and acute myocardial infarction.

### 2.1. PCI for CTO

PCI was performed under local anesthesia through the radial approach as the first choice, either single or dual depending on the initial angiography; as a mixed approach (radial and femoral); or as a bi-femoral approach in cases where the radial artery was not accessible, using arterial sheaths ranging from 6 to 8 French. A dedicated interventional technique was determined for each patient based on the most recent international literature. Procedural success was defined as achieving a TIMI grade of 2 or higher for antegrade flow in all distal branches with a diameter ≥ 2.5 mm and a residual stenosis < 30% at the site of occlusion. Coronary perforation, cardiac tamponade, aortic dissection, periprocedural myocardial infarction [15], complications in non-target vessels, vascular access issues, and the development of contrast-induced nephropathy (CIN) were defined as procedural complications.

### 2.2. Statistical Analysis

Statistical analysis was performed using SPSS statistical software version 23. Continuous variables were expressed as mean ± standard deviation (M ± SD), and categorical variables were expressed as percentages. ANOVA, Chi-squared, and McNemar tests were used to compare continuous and categorical variables, respectively. The statistical significance level for *p*-values was set at 0.05, and confidence intervals (CIs) were set at 95%. The Kaplan–Meier method was used to evaluate survival concerning periprocedural complications and procedural success, as well as the development of MACEs in relation to procedural success.

## 3. Results

The total population of 41 patients consisted of 75.6% male patients, with a mean age of 66.8 ± 8.6 years. The main baseline clinical characteristics of the population are shown in Table 1.

Regarding the main cardiovascular risk factors, hypertension and dyslipidemia were the two most commonly expressed (87.8% and 90.2%, respectively). Additionally, more than half of the population had previously undergone revascularization (56.1% with PCI and 4.9% with coronary artery bypass grafting). For each patient in the population, the indications for CTO PCI (Table 2) and the procedural characteristics (Table 3) were analyzed.

It was observed that in approximately two thirds of the patients, the procedure was guided by the presence of anginal symptoms or equivalents. In 12.2%, the reason for admission was the presence of ischemia detected by stress cardiovascular testing or stress imaging examination. In 17.1% of cases, CTO-PCI was performed due to documented viability in the territory of the target vessel. In one case, the procedure was performed for sustained ventricular arrhythmias in a patient with prior necrosis in the CTO territory, and in the remaining two cases, percutaneous revascularization was performed based on clinical indications, particularly due to the young age of the patient, and the vessel involved was the left anterior descending artery (LAD), despite the absence of symptoms.

The procedure was successful in 80.5% of cases. The primary arterial access used was dual-access, with a preference for radial access with a 7Fr main introducer used in 61% of cases. An anterograde approach was applied in 85.4% of procedures, and the most commonly used technique was wire escalation (WE; 68.3%).

The culprit vessel was in most cases the right coronary artery (58.5%), and PCI was mostly performed as a staged procedure. Major procedural complications occurred in 4 of the 41 patients (9.8%). The detailed percentages of individual complications are reported in Table 4.

No peri-procedural myocardial infarction or major arrhythmic complications were reported. Interestingly, peri-procedural complications occurred only in patients where PCI was successful with optimal vessel recanalization (complications rate in procedural success vs. failure: 24.2% vs. 0%). In 4/41 patients, a femoral artery pseudoaneurysm being treated successfully with ultrasound-guided compression was reported, while only 1 patient developed CIN after the procedure. We evaluated the clinical outcomes of patients who underwent the procedure with a median follow-up of 449 days (interquartile range: 230–643) (Table 5).

**Figure 1:**
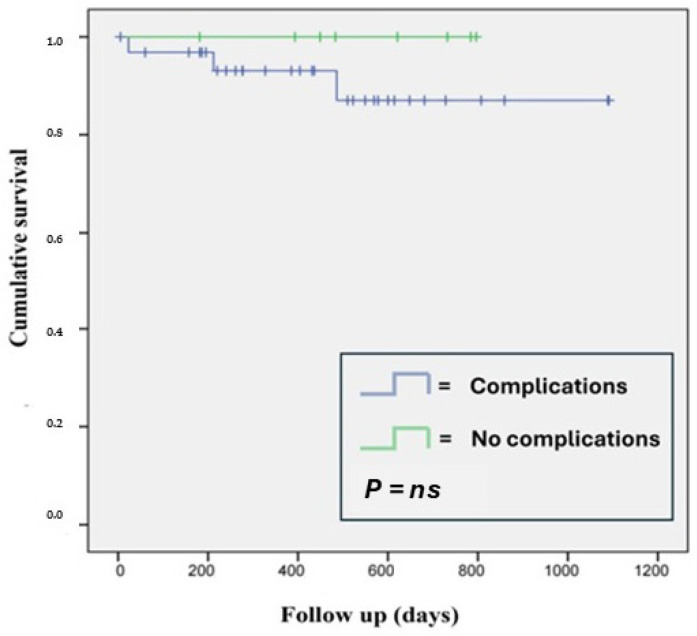
Kaplan–Meier analysis of patient survival at follow-up based on peri-procedural complications.

Of the 41 patients, 3 (7.3%) died after hospital discharge due to non-cardiovascular causes. Kaplan–Meier analysis clearly showed that the occurrence of peri-procedural complications did not affect patient survival (Figure 1).

**Figure 2:**
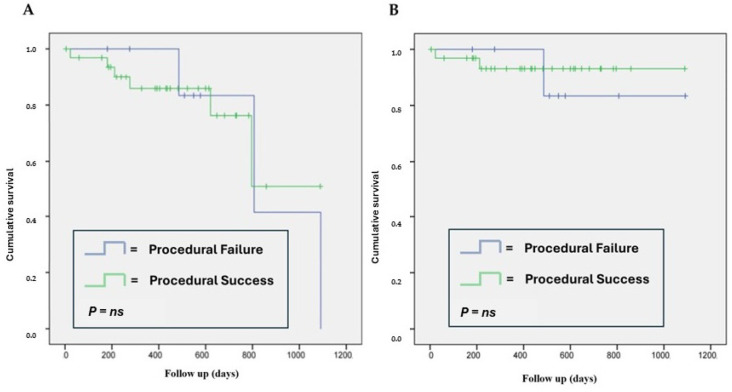
Kaplan–Meier analysis of MACE development at follow-up (**A**) and overall survival (**B**) based on procedural success.

The rate of rehospitalizations for cardiovascular issues was 17.1% (seven patients). Only one patient required repeat PCI of the target vessel, and no cases of stroke or acute myocardial infarction were observed during follow-up. The overall frequency of MACEs was 22.0% (9 patients), and Kaplan–Meier analysis showed that achieving procedural success did not influence the survival rate or MACEs at follow-up (Figure 2).

Finally, regarding the persistence of anginal symptoms or equivalents, the rate was 30.8% (12 patients). Specifically, the percentage of residual angina in patients with procedural success was 29% vs. 37.5% in the group with failed CTO PCI. Furthermore, in the subgroup of patients (*n* = 26) who underwent PCI due to anginal symptoms, the persistence of angina based on procedural success or failure was observed in 33.3% vs. 50.0%, respectively, with *p* = 0.035.

## 4. Discussion

Percutaneous coronary revascularization is currently a safe and effective procedure that, over time, has become the primary method for treating coronary artery disease in both stable and unstable patients, replacing surgical approaches as the main technique for coronary revascularization. A significant factor in the expansion of percutaneous revascularization is the achievement of high success rates by experienced operators, even for procedures as complex as CTO-PCI. Historically, CTO-PCI posed challenges due to having lower success rates compared to conventional procedures, along with a higher incidence of procedural complications and adverse events during follow-up [1]. However, this scenario has evolved in recent years with the development of advanced techniques and specifically designed materials. In high-volume, specialized centers, these advancements have led to reduced complications and success rates nearing those of non-CTO PCI [16,17]. Currently, there are no clear, unified guidelines for managing these patients. Treatment indications are derived primarily from expert consensus, often with conflicting conclusions. The 2018 ESC guidelines [8] recommend CTO-PCI for patients with angina refractory to optimized medical therapy or in the presence of a large ischemic burden in the territory of the occluded vessel. Conversely, the latest American coronary revascularization guidelines [18] suggest that the benefit of CTO revascularization in patients with refractory angina and favorable coronary anatomy remains uncertain (Level 2a B-R). Based on this context and the available literature, we conducted a detailed analysis of patients who underwent CTO-PCI in our institution over the past three years. The aim of the study was to evaluate the appropriateness of indications, procedural efficacy, perioperative complications, and clinical outcomes. The focus on recent years ensures uniformity in treatment indications, technology, and scientific evidence. Our study population aligns with the existing literature, with a slightly lower percentage of male patients and an older average age. The prevalence of major cardiovascular risk factors, such as active smoking, hypertension, and dyslipidemia, was comparably high. However, diabetes mellitus was less prevalent in our cohort compared to the literature (26.8% vs. 43% or 34%) [19,20]. Notably, more than half of the patients (56.1%) had undergone prior percutaneous revascularization, and 4.9% had undergone surgical revascularization. The primary indication for revascularization in our study comprised anginal symptoms (63.4%), consistent with larger studies such as the EuroCTO Trial [21] and the IMPACTOR-CTO Trial [11]. Additionally, we treated patients to reduce ischemic myocardium burden (29.3%) in line with current Europeans guidelines [8]. One patient underwent the procedure for refractory ventricular arrhythmias, achieving symptomatic improvement and reduced arrhythmia burden—a treatment indication that is supported by observational studies [22,23] and is under investigation in the ongoing CTO-ARRHYTHMIA Study [24]. Our procedural success rate was 80.5%, consistent with the existing literature (83.1%, 89.3%) [21,25]. Lesion locations (58.5% RCA vs. 56.7%, 22.0% LAD vs. 23.8%, 19.5% LCx vs. 15.6%) and procedural metrics such as the contrast volume, fluoroscopy time, and radiation dose also aligned with reported data [25]. The anterograde approach was most commonly employed (85.4% vs. ~70% in the literature) [21,25,26], with wire escalation as the preferred technique (68.3%, comparable to 74% in the literature) [27]. The average total stent length was 56.8 ± 33.7 mm, slightly shorter than in reported data (65.9 ± 28.9 mm) [7], reflecting a trend toward reducing stent usage to minimize prolonged dual antiplatelet therapy (DAPT), particularly in older patients. A hybrid approach, combining stents and DEB, was favored, limiting stenting to the primary lesion and treating the remaining vessel with DEB. In our population, no significant hemorrhagic or thrombotic complications were observed at follow-up that would demonstrate, at least within the available follow-up period, a documented benefit from the use of a hybrid strategy. Overall, while not strongly validated in the literature, this strategy appears promising for complex revascularizations such as CTO-PCI [28].

We observed a procedural complication rate of 9.8%, which is higher than the literature reports (4.7%) [25], likely due to our smaller sample size and broader complication definitions. Coronary perforation was noted in 2.4% of cases, consistent with the literature (2.9%) [29]. Notably, there were no periprocedural myocardial infarctions, and the contrast-induced nephropathy incidence was 2.4% (vs. 3.4% in the literature) [30]. Unlike in the literature, where procedural failure was associated with a doubled rate of complications [25], in our cohort, procedural failure was not associated with complications, possibly reflecting conservative operator decision-making based on risk–benefit evaluations. The observed global mortality rate was 7.3%, and MACEs occurred in 22% of patients, primarily in those with procedural failure (18.2% vs. 37.5%). These findings and the significative reduction in symptoms in the subgroup of symptomatic patients after an effective procedure highlight the benefits of a successful CTO-PCI in improving quality of life and reducing cardiovascular events compared to medical therapy alone. Rehospitalization rates for cardiovascular causes (17.1%) and target vessel re-PCI rates for in-stent restenosis (2.4%) were comparable to the literature data [31]. Anginal symptoms persisted in 29% of patients with successful procedures and 37.5% with failed procedures in the global population, but regarding just symptomatic patients, the statistical spread increased, reaching significance (33.3% vs. 50.0%; *p* = 0.035). These findings align with the EuroCTO and IMPACTOR-CTO Trials [11,21]. It should be noted that another study, the DECISION-CTO Trial [12], did not demonstrate any difference between the use of percutaneous revascularization and optimal medical therapy in symptom control, highlighting the ongoing lack of solid and consistent data on this issue.

## 5. Limitations

The main limitation of our study is that it is a single-center study with a small sample size, partly due to the restricted evaluation period being limited to the last three years. While this is a limitation, it also allowed us to analyze a population as homogeneous as possible in terms of indications and treatment types, following the most recent scientific evidence and advanced technologies available. The observational and retrospective nature of the study may have introduced a selection bias regarding the complexity of the patients and their coronary profiles. Furthermore, for the same reason, it was not possible to retrieve all the potentially relevant data for the study or to compare the outcomes with those in a population of patients with CTO treated conservatively. Lastly, the recent introduction of DEB in CTO PCI further reduced the number of patients in this subgroup, making additional ad hoc analyses impossible.

## 6. Conclusions

Our study yielded results consistent with the literature (REF), allowing us to affirm that the percutaneous revascularization of a CTO has a high success rate and a low incidence of major complications, making it a viable therapeutic option in addition to optimized medical therapy. Moreover, the CTO PCI procedure improves quality of life by reducing angina symptoms and the incidence of MACEs at follow-up. Careful patient selection, based on the assessment of angina symptoms or significant ischemic areas, enables a long-term benefit superior to that of optimized medical therapy alone. The increased use of DEB in this procedural context could further reduce the total stent length implanted, thereby decreasing the patient’s long-term thrombotic risk and minimizing the hemorrhagic complications associated with prolonged dual antiplatelet therapy. Future or ongoing randomized studies will help clarify the optimal management of these patients.

## Figures and Tables

**Table 1:** Clinical characteristics of the population.

Age (years)	66.8 ± 8.6
Male sex (*n*) %	(31/41) 75.6
Hemoglobin (g/dL)	13.4 ± 1.4
LDL cholesterol (mg/dL)	76.1 ± 37.6
Creatinine (mg/dL)	1.3 ± 0.9
Active smoker (*n*) %	(21/41) 51.2
Family history of CAD (*n*) %	(20/41) 48.8
Diabetes (*n*) %	(11/41) 26.8
Hypertension (*n*) %	(36/41) 87.8
Dyslipidemia (*n*) %	(37/41) 90.2
Known CAD (*n*) %	(31/41) 75.6
Previous PCI (*n*) %	(23/41) 56.1
Previous CABG (*n*) %	(2/41) 4.9
Ejection fraction (*n*) %	49 ± 8
Anticoagulant (*n*) %	(4/41) 9.8
Acetylsalicylic acid (*n*) %	(31/41) 75.6
Beta blockers (*n*) %	(29/41) 70.7
Nitrates (*n*) %	(3/41) 7.3
Ranolazine (*n*) %	(4/41) 9.8
Ivabradine (*n*) %	-

LDL, low-density lipoprotein; CAD, coronary artery disease; PCI, percutaneous coronary intervention; CAGB, coronary artery bypass graft. Data are expressed as mean ± standard deviation (M ± SD) or percentage (%).

**Table 2:** Procedural indications.

Indication	% (*n*)
Angina	63.4 (26)
Instrumental ischemia	12.2 (5)
Viability	17.1 (7)
Ventricular arrhythmias	2.4 (1)
Other indications	4.9 (2)

**Table 3:** Procedural characteristics.

**Procedural success** (%)	80.5
**Arterial access** (%)	
Double	70.7
Single	29.3
**Access type** (%)	
Only radial	65.9
Radial and femoral	31.7
Only femoral	2.4
**IABP** (%)	4.9
**Sheat (higher dimension)**	
6F	29.3
7F	61
8F	9.8
**Culprit lesion** (%)	
LAD	22
LCx	19.5
RCA	58.5
**CTO anterograde** (%)	85.4
**CTO-PCI technique** (%)	
WE	68.3
Reverse CART	12.2
Carlino	2.4
DR	17.1
**Total stent** length (mm) (M − SD)	56.8 ± 33.7
**DEB** (%)	22
**Contrast media** (mL) (M − SD)	221.3 ± 112.6
**Fluoroscopy time** (min) (M − SD)	42.6 ± 29.4
**Radiation dose** (mGy/cm^2^) (M − SD)	2338.6 ± 1556.8

IABP, intra-aortic balloon pump; LAD, left anterior descending artery; LCx, left circumflex artery; RCA, right coronary artery; WE, wire escalation; reverse CART, reverse controlled anterograde and retrograde tracking; DR, dissection and re-entry.

**Table 4:** Procedural complications.

Complication	Percentage (*n*)
Coronary perforation	2.4 (1)
Cardiac tamponade	2.4 (1)
Aortic dissection	2.4 (1)
Complication non-target vessel	2.4 (1)

**Table 5:** Clinical outcomes.

Outcome	Percentage (*n*)
Death	7.3% (3)
Residual angina	30.8% (12)
Re-PCI	2.4% (1)
Stroke	0 (0)
AMI	0 (0)
Re-hospitalization	17.1% (7)
MACEs	22.0% (9)

PCI, percutaneous coronary intervention; AMI, acute myocardial infarction; MACEs, major adverse cardiac events.

## Data Availability

Data were collected in a dedicated password-protected database. The study was conducted in accordance with the Declaration of Helsinki and all patients signed an informed consent form regarding the treatment of personal data.
